# Modern experimental methods for assessing the effectiveness of tissue-engineered products for hyaline cartilage regeneration

**DOI:** 10.3389/fbioe.2025.1595116

**Published:** 2025-07-21

**Authors:** M. S. Bozhokin, Yu. S. Korneva, S. A. Bozhkova, E. R. Mikhaylova, D. M. Marchenko, B. R. Rakhimov, Y. A. Nashchekina, M. G. Khotin

**Affiliations:** ^1^ Center of Cell Technologies, Institute of Cytology Russian Academy of Science, Saint-Petersburg, Russia; ^2^ Centre of Traumatology and Orthopedics, Department of Wound Infection, Vreden National Medical Research, Saint-Petersburg, Russia; ^3^ North-Western State Medical University named after I.I. Mechnikov, Saint Petersburg, Russia; ^4^ International Laboratory of Bioinformatics, Faculty of Computer Science, HSE University, Moscow, Russia

**Keywords:** tissue engineering, methods, review, hyaline cartilage, recovery

## Abstract

Hyaline cartilage (HC) is a specialized connective tissue that covers the surfaces of major joints and is characterized by its limited regenerative capacity. Modern therapeutic approaches to HC restoration often do not provide complete regeneration of damaged tissue. Developed tissue engineering methods show promise as effective approaches for restoring various types of HC damage. Due to the rapid evolution of various technologies in research practice, the range of methods available for analysis of TE constructs has expanded, including for the study of tissue engineering of hyaline cartilage (TEHC). Because of the complexity of the HC’s structure, a whole range of methods is needed to assess characteristics of the scaffold, such as structure and strength. It is also important to study the behavior of cells inside the TE construct at all stages of cultivation, including post transplantation into the damaged area. The opacity of the scaffold and the complexity of its architecture often cause issues with the cell visualization and assessment of their viability. Therefore, there is a need to optimize each specific method for each specific scaffold. Despite the active study of TEHC, the results remain unsatisfactory. In this study, we have systematized data on the effectiveness and feasibility of methods to analyze structure, mechanical characteristics, cell interaction with the scaffold, and their ability to form new tissue before and after transplantation.

## Introduction

The goal of this study is to analyze experimental techniques dedicated to research tissue engineering of hyaline cartilage (TEHC). The concept of tissue engineering, proposed by Langer (Langer and Vacanti, 1993) in 1993, involves the development of–Cell-engineered construct CECs based on biodegradable scaffolds, cell cultures, and chemical patterns for modulating cell proliferation or hyaline cartilage recovery. The article describes methods for evaluating different stages of research in tissue engineering of hyaline cartilage, comparing their advantages, disadvantages, areas of application, and the final results achieved.

## Materials and methods

### Literature search and selection criteria

**FIGURE 1:**
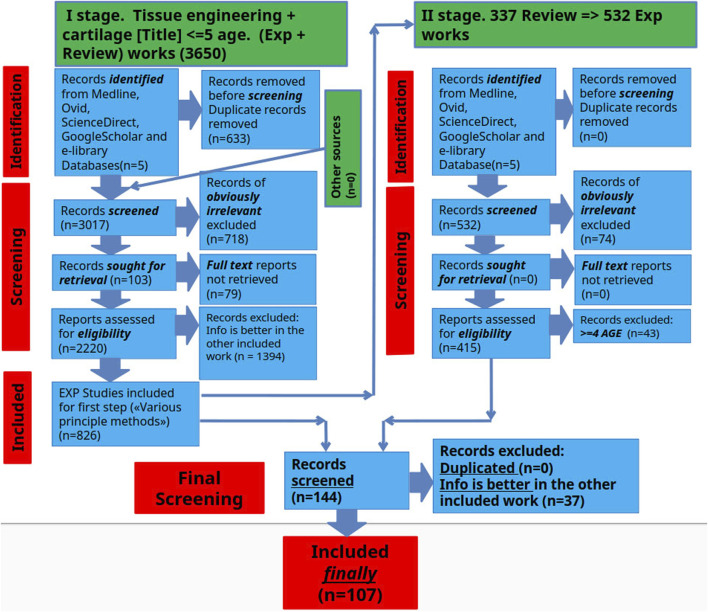
Schematic representation of the selection process for scientific articles included in this review.

A literature review was conducted to identify existing methods for assessing the effectiveness of tissue engineering of hyaline cartilage in PubMed (MEDLINE), eLIBRARY, ScienceDirect, and Google Scholar databases, retrieving literature available up to mid-2024. The article reviews original works devoted to various methods for assessing the effectiveness of hyaline cartilage tissue engineering. The search was conducted in two stages. In the first stage, we analyzed the articles and searched for fundamental methods for assessing the effectiveness of hyaline cartilage tissue engineering. In the second stage, we deepened the search and detailed the information on each of these methods. Studies were included if they simultaneously met the following criteria: (1) included experimental hyaline cartilage tissue engineering 2) included a performance evaluation method 3) were in open access. Studies were excluded if 1) the full text was not available 2) there was no connection with hyaline cartilage 3) there was no connection with tissue engineering 4) there were no methods to evaluate the experimental performance 5) the work was clinical and did not contain experimental data with animals. Subsequently, experimental studies were examined, and relevant review articles in the field were used to identify additional literature for further analysis. Experimental studies from the past 5 years were prioritized. In parallel, the searches focused on specific methods and their applications in research tissue engineering of hyaline cartilage were carried out (Figure 1).

### Study selection process

The search results underwent a rigorous screening process to identify and eliminate duplicates according to the predefined inclusion criteria. This assessment aimed to select the articles that would ultimately be included in the final information extraction. The screening involved 6 independent reviewers working in pairs who carefully assessed the titles, abstracts and full texts of the manuscripts during two separate screening phases.

### Data collection

The included articles were added to a spreadsheet. The selected studies were categorized according to the principle of the method. The authors did not aim to analyze all publications currently available on this topic. However, we believe that the adopted search and analysis strategy ultimately achieved the primary objectives of the study: to consolidate data on research methodologies and to evaluate their advantages and disadvantages. It is important to note that the authors define a “research method” as a “block and set of specific techniques for evaluating the effectiveness of tissue engineering of hyaline cartilage in a particular area of study.”

## Microscopic studies

**FIGURE 2:**
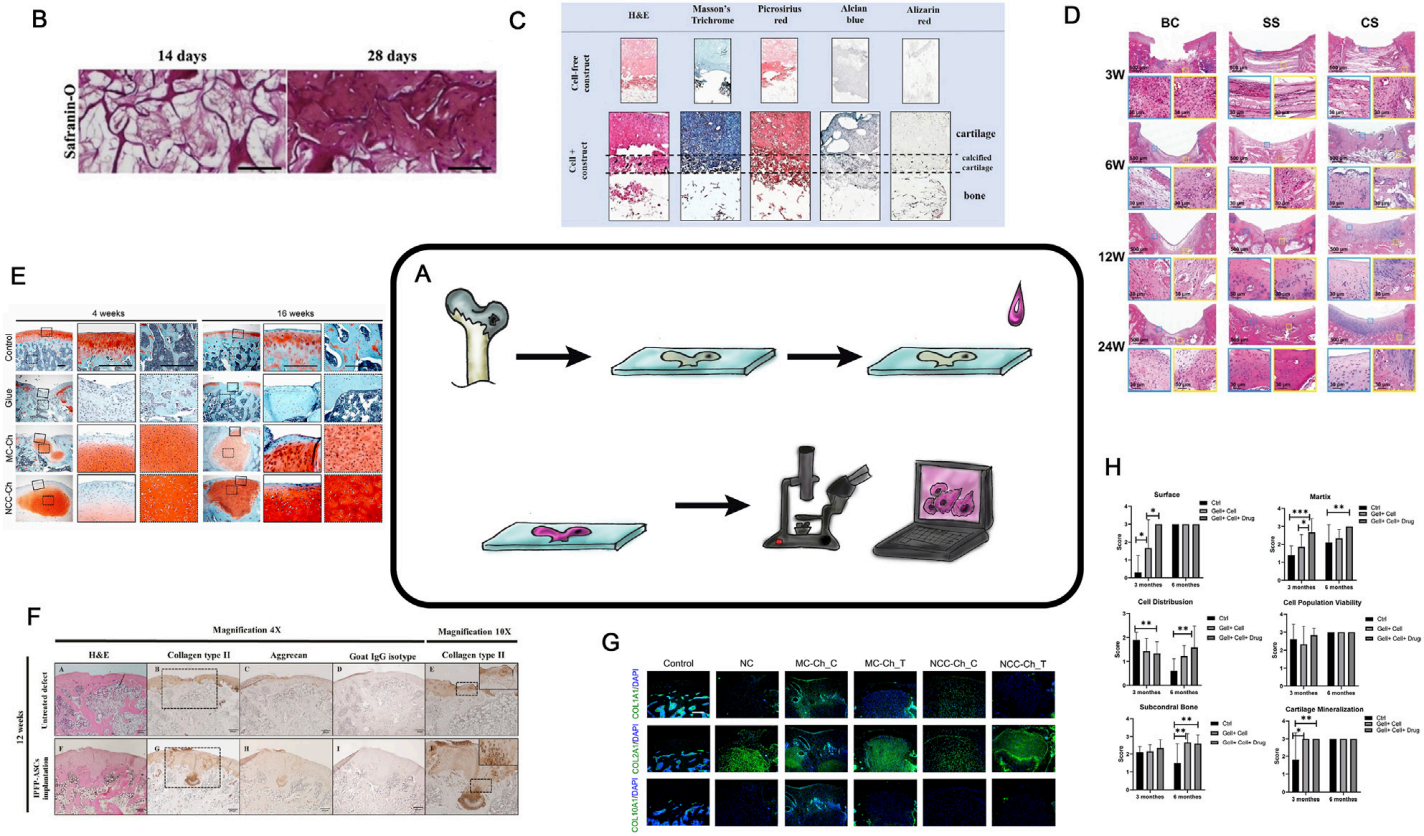
Histological analysis in TEHC. **(A)** is a schematic diagram of a histological examination. **(B)** Safranin-O staining of BMSCs in the FC-CS scaffolds for *in vitro* 14 or 28 days. Taken from the article (Nikolai et al., 2020; Zhou et al., 2020). **(C)** Histological examination of themulti-layered osteochondral scaffolds after 21 days of co-culture. The paraffin mounted scaffolds were sectioned and stained with hematoxylin and eosin **(H,E)**, Masson’s trichrome and Picrosirius red for total collagen, Alcian blue for GAGs and Alizarin red for mineralization. Cell-free scaffolds were also stained as control. Taken from the article (Zhou et al., 2020; Korpayev et al., 2020). **(D)** Microscopic appearance of H&E-stained blank control, simple scaffold, and composite scaffold groups after 3, 6, 12 and 24 weeks. BC, Blank Control; SS, Simple Scaffold; CS, Composite Scaffold; H&E, hematoxylin and eosin. Taken from the article (Han et al., 2021a). **(E)** Rat joints analyzed by Safranin O/Fast Green staining. Solid boxed (superficial) and dashed boxed (subchondral bone) areas in the left column are shown at a higher magnification in the central and right column, respectively. Taken from the article (Lee et al., 2021). **(F)** Representative histological results obtained from the untreated defect and implanted IPFP-ASCs group at 4 weeks post-operation. H&E staining **(A,F)**, immunohistochemistry staining for type II collagen **(B,G)**, aggrecan **(C,H)**, and goat IgG isotype as a negative control **(D,I)**. Magnification 4×. Scale bars at 200 μm as indicated. Higher magnifications of both groups were enlarged from the black dotted square in the images B and G, respectively **(E,J)**. Magnification 10×. Scale bars at 100 μm as indicated. Taken from the article (Sriwatananukulkit et al., 2022). **(G)** Immunofluorescence staining of regenerated cartilage for detection of COL1A1, COL2A1, and COL10A1. Nuclear DNA was labeled with DAPI Taken from the article (Lee et al., 2021). **(H)** Results of *in vivo* cartilage defect repair: International Cartilage Repair Society (ICRS) scoring of the gross appearance of the regenerated cartilage; n = 12, Taken from the article (Naghizadeh et al., 2021).

Among the methods used to assess the effectiveness of tissue engineering of hyaline cartilage, histological and/or microscopic studies hold a central position. This basic method requires specialized skills; however, it is economically affordable. Despite their two-century history (Karamanou et al., 2010) microscopy remains one of the most widely used and unbiased methods for evaluating experimental outcomes (Figure 2A). Microscopy is widely applied in *ex vivo* experiments to assess cell status within cultures and CECs during cultivation stages and to monitor cell proliferation and aggregation during modifications or enhancing extracellular. Matrix (ECM) synthesis (Figure 2B) (Nikolai et al., 2020). Cross-sections of cell-engineering constructs are stained with various dyes also to analyze cell proliferation and protein synthesis within designated zones (Figure 2C) (Zhou et al., 2020; Korpayev et al., 2020). Standard *in vivo* experiments histological analysis involves sample fixation and preparation of tissue sections containing the region of interest. (Figure 2C) (Zhou et al., 2020; Korpayev et al., 2020).

In some cases, special microscopic films are used for the preparation of sections from hard samples such as in this works (Morodomi et al., 2019; Bozhokin et al., 2021a).

Special attention should be paid to consistent 3D positioning of the sample before microtomy (Han et al., 2021a; Al-Sabah et al., 2019) (Figure 2D). The selection of fixation and decalcification protocols as well effects the state and parameters of the regenerated area, making the preanalytical stage of research critically important (Király et al., 1996). To determine the precise localization of various ECM proteins within the structural components of TEHC, histochemical analysis with stains specific to the protein properties can be also employed (Lee et al., 2021; Sriwatananukulkit et al., 2022) (Figures 2E,F). Currently, good practice declares that all images are processed to yield numerical parameters, such as ECM quantity, cellular morphology or calcification (Figure 2H) (Naghizadeh et al., 2021)*.* Histochemical analysis can be performed on culture plastic or glass, while evaluating chondrogenic differentiation *in vitro* (Albert and Creech, 1941 ; Bozhokin. et al., 2021a). For detailed information on chondrogenesis, confocal microscopy is used (Figure 2G) (Lee et al., 2021). And so it is possible to assess the chondrocyte viability in their natural 3D arrangement or capture the images at different depths within native tissue or cell-engineering construct (Al-Sabah et al., 2019; Galarraga et al., 2021).

Microscopic tissue analysis results can be quantitatively evaluated using various scoring systems, based on staining intensity or ratio of stained structures or cells to total area or nuclear count (O’Driscoll et al., 1986). Images, that are evaluated using histological scoring systems (International Cartilage Repair Society grading system (ICRS), O'Driscoll), can conclude the overall experiment’s effectiveness (Chen et al., 2020; Murata et al., 2022; Sun et al., 2021; Guo et al., 2021). Unfortunately, all “semi-quantitative” histological evaluation systems are observer-dependent. In 1994, it was proposed an automated cartilage assessment based on color differences in safranin-O-stained specimens. Modern software for histological image analysis can automatically calculate numerous histological parameters (specific cell types, amounts of ECM proteins, the area of defect filling by the regenerate, and many others) with minimal time investment (Farshid Moussavi-Harami et al., 2009; Yang et al., 2019; Rutgers et al., 2010). Specialized software or scoring systems help to transform histological research results from subjective qualitative assessments to statistically significant numerical parameters. The application of AI in the analysis of histological preparations is developing dynamically. For example, in the article (Nagarajan et al., 2024) authors use an algorithm that involves evaluating histological images (e.g., safranin O staining and chondrocyte distribution) using automatic classification methods based on artificial intelligence (such as deep learning). The progression of such work over the last few years is significant; while in 2022 such software could automatically recognize individual elements on histological images (such as the lateral and medial condyles (Mori et al., 2022)), in 2023 the software learned to automatically assess the degree of OA from histological preparations (Khader and Alquran, 2023), by 2024 AI-based software was already reliably and objectively assessing the degree of hyaline cartilage repair. In the authors’ view, such tools will continue to improve and we could see an explosive growth in such work in the near future.

At present, microscopic studies in tissue engineering of hyaline cartilage are the most important reference methods by which the effectiveness of the whole experiment can be unequivocally assessed, without which no modern scientific article is published. In the near future, in our opinion, online tools using AI to evaluate experimental images using a unified algorithm are likely to appear which will greatly simplify the comparison of studies performed by different teams.

## Flow cytometry

**FIGURE 3:**
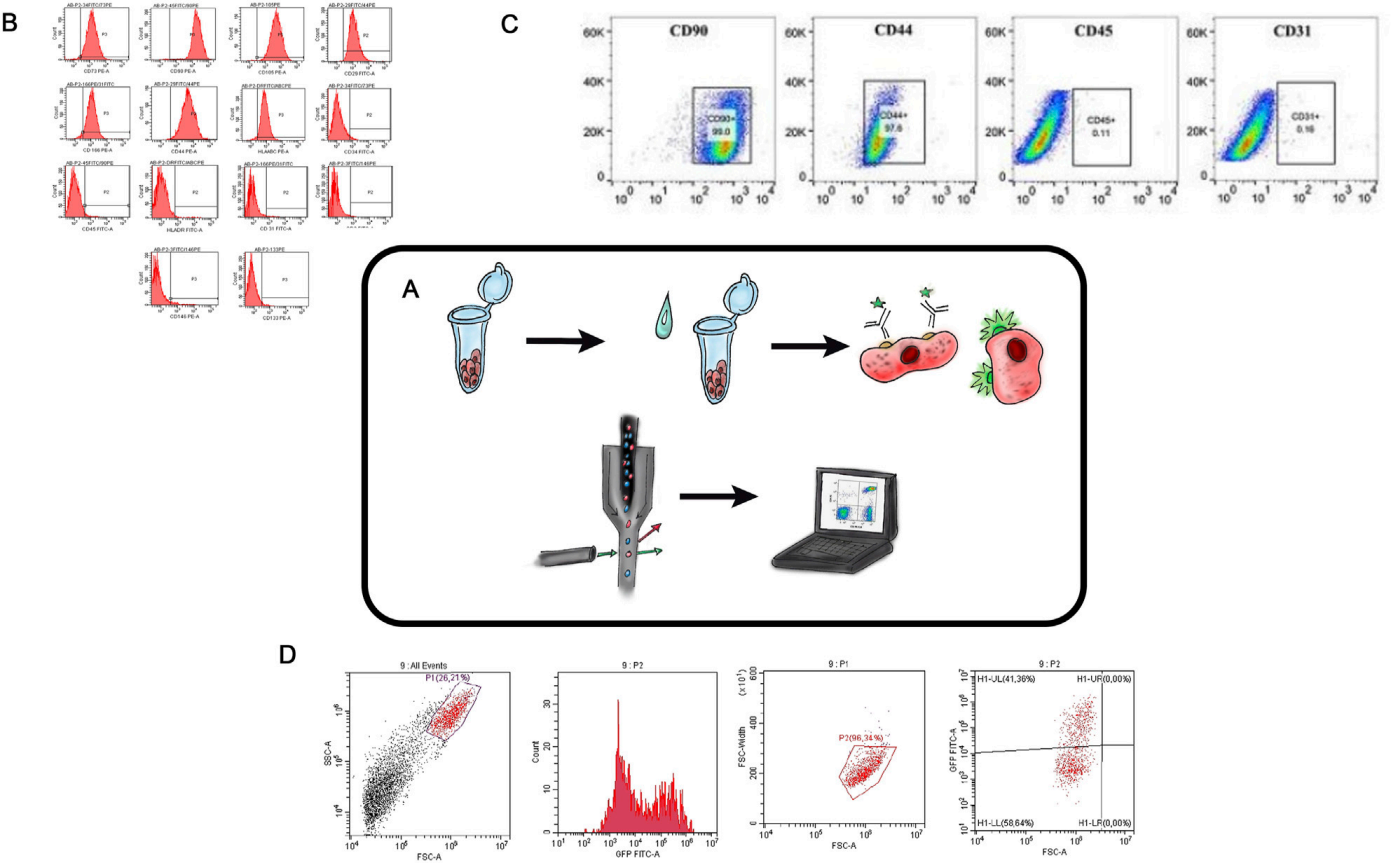
Flow Cytometry in TEHC **(A)** – Schematic diagram of flow cytometry. **(B)** – Collective flow cytometry histograms for mesenchymal stem cells markers (CD105, CD44, CD166, CD29, CD90, and CD73 and HLA-ABC) and antibodies specific to haematopoietic cells (CD45, CD34, CD14 and HLA-DR) were displayed for the hASCs retrieved from the confluent cultures at the 7th day of passage 2. Areas in red color indicate stained cells. hASC, human adipose-derived stem cell. (For interpretation of the references to color in this figure legend, the reader is referred to the web version of this article. Taken from the article (Piagnerelli et al., 2007). **(C)** – FCM analysis of the expression of stem cell identification-related antibodies in rASCs. Taken from the article (Liu et al., 2022a). **(D)** – Cytofluorometric analysis of human dermal fibroblasts with lentiviral transduction *Tgfb3* gene (authors unpublished data).

Flow cytometry (FC) is a modern technology that enables rapid, multiparametric analysis of individual cells in an automated manner (Figure 3A) (Moldavan, 1934; Gucker et al., 1947; Walles, 1956). The method is based on detecting fluorescence and light scattering (i.e., physically and biologically determining antigens on different cell types and inside the cell bodies) (Figure 3B). Quantitative characteristics of cell populations used in the TEHC projects include cell shape, proliferative and clonogenic potentials, immunological profile, morphology, phenotype (Piagnerelli et al., 2007), size (Jerald et al., 2002), viability (Ouyang et al., 2019; Rasouli et al., 2003), nucleic acid content (Lebaron et al., 2002), and intracellular processes (Darzynkiewicz et al., 2001; Piotr and Darzynkiewicz, 2004). Additionally, this method can be used to assess cell aggregation, native fluorescence, expression of surface markers, and last but not least, the cells can be sorted (Figure 3C) (Rasouli et al., 2003; Zhao et al., 2018; Jolene and Bradford, 2011).

FC was used to determine the immunophenotypic profile of adipose stem cell (ASC) by analyzing the presence of mesenchymal stem cell surface markers connected to the cell-engineering constructs development (Tulin, 2020) (Figure 3B). In another study (Liu et al., 2022) the identification of chondrogenic, osteogenic, and adipogenic differentiation potentials of stem cells derived from rat adipose tissue (Figure 3C) was performed. Flow cytometry kits are now available on the market, and so the determination of immunophenotypic profiles, proliferative capacities, and DNA content assessment for different types of cells can be done simultaneously and with minimal activity (Jolene and Bradford, 2011). Flow cytometry is an efficient and precise method for evaluating the effectivity of genetic cell modifications, especially when fluorescent genes are inserted in the expression plasmid (Figure 3D) (Bozhokin et al., 2021c). A crucial practical application of FC lies in its ability to sort cells into distinct subpopulations. For example, Chen-Shuang Li investigated the chondrogenic differentiation potential of human perivascular stem cells under the influence of a combination of growth factors that were previously isolated from the human stromal vascular fraction using fluorescence-activated cell sorting (FACS) (Li et al., 2016).

An important consideration for cell-engineering constructs materials is their biocompatibility with the native tissue microenvironment and with proliferating cells. In a study which aimed to create a composite hydrogel for an HC defect repair, a composite hydrogel based on strontium alginate was compared with a strontium alginate/chondroitin sulfate composite hydrogel (Ma et al., 2019). An MTT assay was used to determine the cytotoxicity of the material and its ability to support chondrocyte proliferation, while FC was used to assess apoptosis levels in chondrocytes and the viability of cell populations depending on the hydrogel material used.

FC has extensive potential applications in studies aimed at developing new tissue engineering of hyaline cartilage approaches. The advantages of flow cytometry in this field include the ability to simultaneously analyze a large number of cells with minimal time investment, as well as the automated evaluation of cell viability and proliferative activity. FC can be used to assess the effectivity of TE constructs modifications based on fluorescent signal levels or the expression of specific surface markers. Additionally, FC enables cell sorting into distinct subpopulations, which may have varying potentials for tissue regeneration and chondrogenic differentiation. Thus, flow cytometry is an easy-to-use, economically available numerical technique for evaluating various cell subpopulations and cell modification methods.

## Reverse transcription - Polymerase chain reaction

**FIGURE 4:**
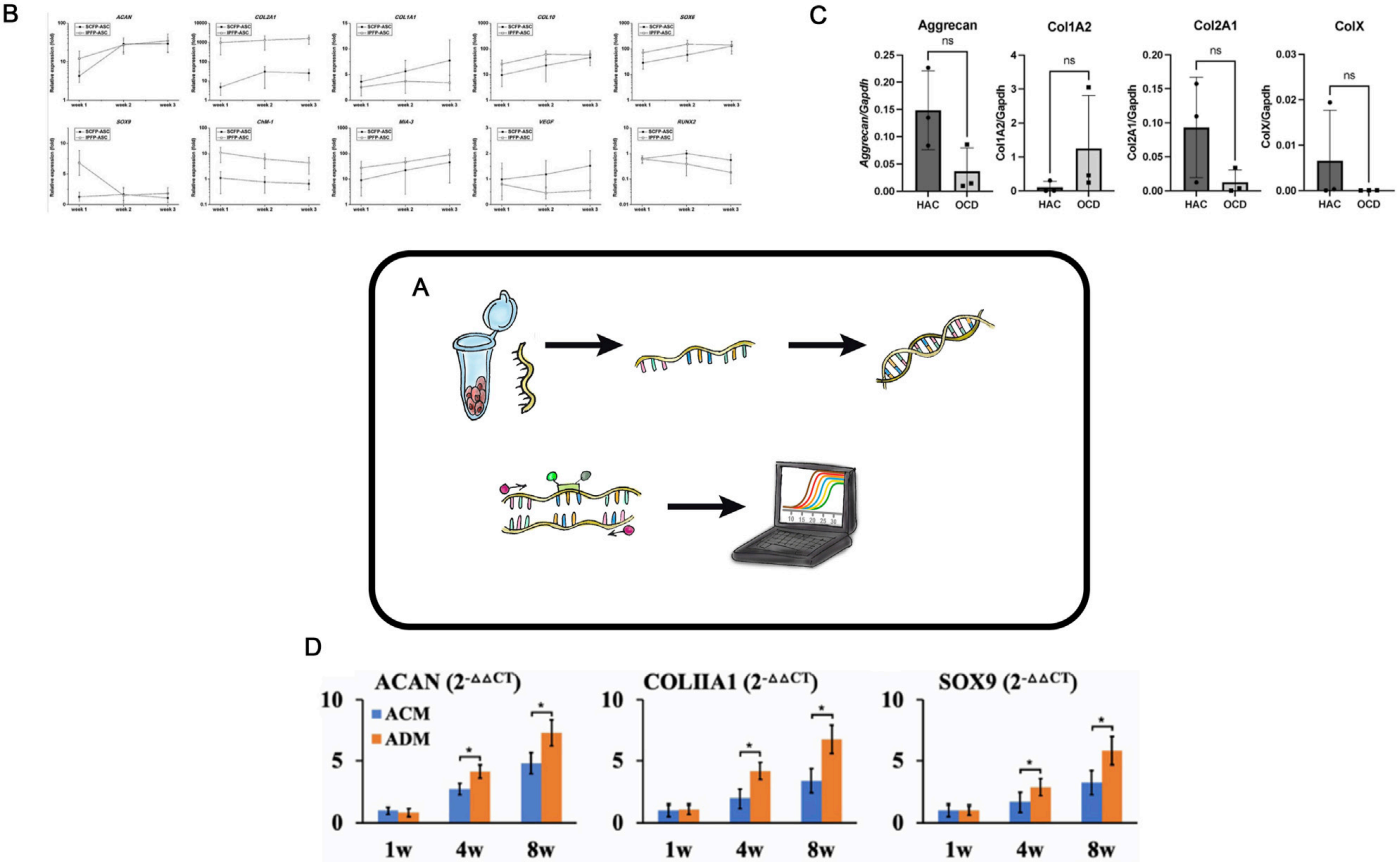
Reverse transcription PCR in TEHC **(A)** – Schematic representation of the real-time polymerase chain reaction (PCR) in hyaline cartilage tissue engineering. **(B)** – mRNA expression profile of the ASC-derived chondrocytes in 3D matrix. The IPFP-ASC-derived chondrocytes had higher ACAN mRNA expression than did the SCFP-ASC-derived chondrocytes at week 1 and extremely high COL2A1 expression. By contrast, the SCFP-ASC-differentiated chondrocytes exhibited significantly higher COL1A1 expression at weeks 2 and 3. Although the IPFP-ASC-derived chondrocytes had high COL10 level at weeks 1 and 2, they also had significantly higher SOX6 (weeks 1 and 2), SOX9 (week 1), ChM-1 (weeks 1, 2, and 3), and MIA-3 (weeks 1, 2, and 3) levels and lower VEGF (weeks 2 and 3) and RUNX2 (weeks 2 and 3) levels than the SCFP-ASC-derived chondrocytes in 3D matrix. ASCs, adipose tissue–derived stem cells; IPFP, infrapatellar fat pads; SCFP, subcutaneous fat pads. Taken from the article (Wang et al., 2021a). **(C)**– Real-time PCR analyses of OCD chondrocytes and healthy articular chondrocytes. Three OCD and HAC cartage donors were analyzed for expression of Aggrecan, Collagen type I (Col1A2); Collagen type II (Col2A1); and Collagen type X (ColX). Ns no statistically significant difference. The CT values were normalized to GAPDH housekeeping gene. All assays were performed in triplicates. Taken from the article (Vapniarsky et al., 2022). **(D)**– QPCR analysis of in vitro ECs in ACM and ADM groups. Expression of ACAN, COLIA1 , and SOX9 genes in ACM and ADM groups after 1, 4, and 8 weeks of in vitro culture. *P < 0.05. Taken from the article (Wang et al., 2021b).

The classical polymerase chain reaction. (PCR) method was introduced by Kary Mullis in 1983 (Mullis, 1990). Reverse transcription PCR (RT-PCR) has broad applications in various fields, such as disease diagnosis, virus genotyping (Paulina Rajko-Nenow and Batten, 2022), detection and quantification of microorganisms in food products (Kingsley et al., 2010), studies of gene expression changes during cellular processes, pathological conditions, wound healing, and many more. It is also actively and routinely used in tissue engineering of hyaline cartilage (Figure 4A) (Ma et al., 2019; Nour-Eldeen et al., 2020).

In tissue engineering of hyaline cartilage, RT-PCR is used to evaluate changes in the relative expression of key chondrogenesis genes, including *Col2A1, Col1A1, ACAN*, *Sox9*, *TGF-β3*, and *Comp* (Figures 4B–D). The advantages of RT-PCR are its simplicity, cost-effectiveness, and the quantitative nature of results, making RT-PCR particularly suitable for the initial screenings. In a study by Ye Sun aimed at developing a CEC containing growth differentiation factor 5 and bone marrow-derived stem cells (Sun et al., 2019), RT-PCR was used to compare marker gene expression levels across experimental groups. In another work, allogenic chondrocytes were transplanted using a hybrid scaffold made of chitosan hydrogel and demineralized bone matrix to repair rabbit cartilage defects (Chen et al., 2016). RT-PCR analysis revealed increased mRNA levels of insulin-like growth factor 1, bone morphogenetic protein 7, and hepatocyte growth factor 1 month after transplantation, and so indicating activation of these genes.

RT-PCR is widely used in tissue engineering of hyaline cartilage research to select the optimal matrix for constructs, identify the best cell donors, or choose cell cultures with specific chondrogenic differentiation parameters. For example, chondrocytes isolated from various tissue regions can exhibit these various chondrogenic differentiation potentials when cultured in 3D scaffolds. In a study (Wang et al., 2021a), the expression profile of mRNA in ASCs cultured in a gelatin-based 3D scaffold was analyzed using RT-PCR, which helped to identify the most suitable cell source for TEHC (Figure 4B). In another study, authors used RT-PCR to evaluate the expression of key chondrogenesis genes in chondrocytes isolated from cartilage fragments of donors with osteochondritis dissecans compared to healthy donors (Figure 4C) (Vapniarsky et al., 2022). RT-PCR is widely used to select the most suitable scaffold for creating tissue engineering of hyaline cartilage constructs: Wang (Wang et al., 2021b) employed this method to compare the expression of some genes and demonstrated that an acellular cartilage matrix was superior to an acellular dermal matrix (Figure 4D).

Many researchers in tissue engineering of hyaline cartilage intentionally manipulate the proliferation and differentiation of cells used in CECs, modifying them via various methods to enhance their effectiveness. RT-PCR is essential in such studies where cell modification is used and where researchers evaluate the resulting chondrogenic differentiation of cells. RT-PCR is rapid and economically accessible method; it provides quantitative data to evaluate cell proliferation and chondrogenic modification, cytotoxicity and biocompatibility of matrices and scaffolds, underscoring its necessity for the primary analysis of novel tissue engineering of hyaline cartilage techniques.

## RNA-seq analysis

A further development of RT-PCR method is RNAseq analysis. This method is employed to ascertain the palette and expression profiles of a variety of genes in a cell culture or subpopulation of cells. However, it is expensive and requires mandatory subsequent bioinformatic analysis. This approach is preliminary BEFORE directly experimenting with tissue engineering of hyaline cartilage. It takes quite a lot of effort to direct this tool to solve practical experimental problems.

A paucity of research has been found on the use of RNAseq analysis in tissue engineering of hyaline cartilage, which can be attributed to the complexity and economic cost of the experiment. The primary objective is to determine the most effective method of cartilage regeneration. Cell modification, encompassing the analysis of gene expression changes, constitutes a secondary yet equally significant undertaking. In article 2025, an injectable hydrogel for cartilage regeneration was investigated, and an increase in the expression of genes responsible for hyaline cartilage metabolism was shown by RNA-seq method (Zhou et al., 2025). Utilizing this methodology in study of the application of ascorbic acid to costal chondrocytes made it possible to precisely determine the alteration in gene expression profile and observe potential osteogenic differentiation and cartilage hypertrophy (Zheng et al., 2024). The method also allows to clarify which cell types are affected in OA, which gene networks regulate OA progression, and which there are cell subtypes are present in hyaline cartilage at different stages of OA. (Gu et al., 2023). Currently, this technique allows for more accurate and efficient selection of a specific cell line for use in tissue engineering of hyaline cartilage (Jiang and Tuan, 2015). Another potential use of this technique is the preliminary analysis of cell culture by scRNA-seq of banked and already described cell culture. (Gu et al., 2023). However, the impact of the methodology and its potential for implementation in tissue engineering of hyaline cartilage is still indirect. The methodology is complex, economically unprofitable and requires solving a large number of technical and computational problems. However, it is worth noting that it is possible to use AI learning technologies to help with the decoding of the data obtained and thus, perhaps, one of the difficulties of using this method will be solved (Gu et al., 2023).

## Proteomic analysis

**FIGURE 5:**
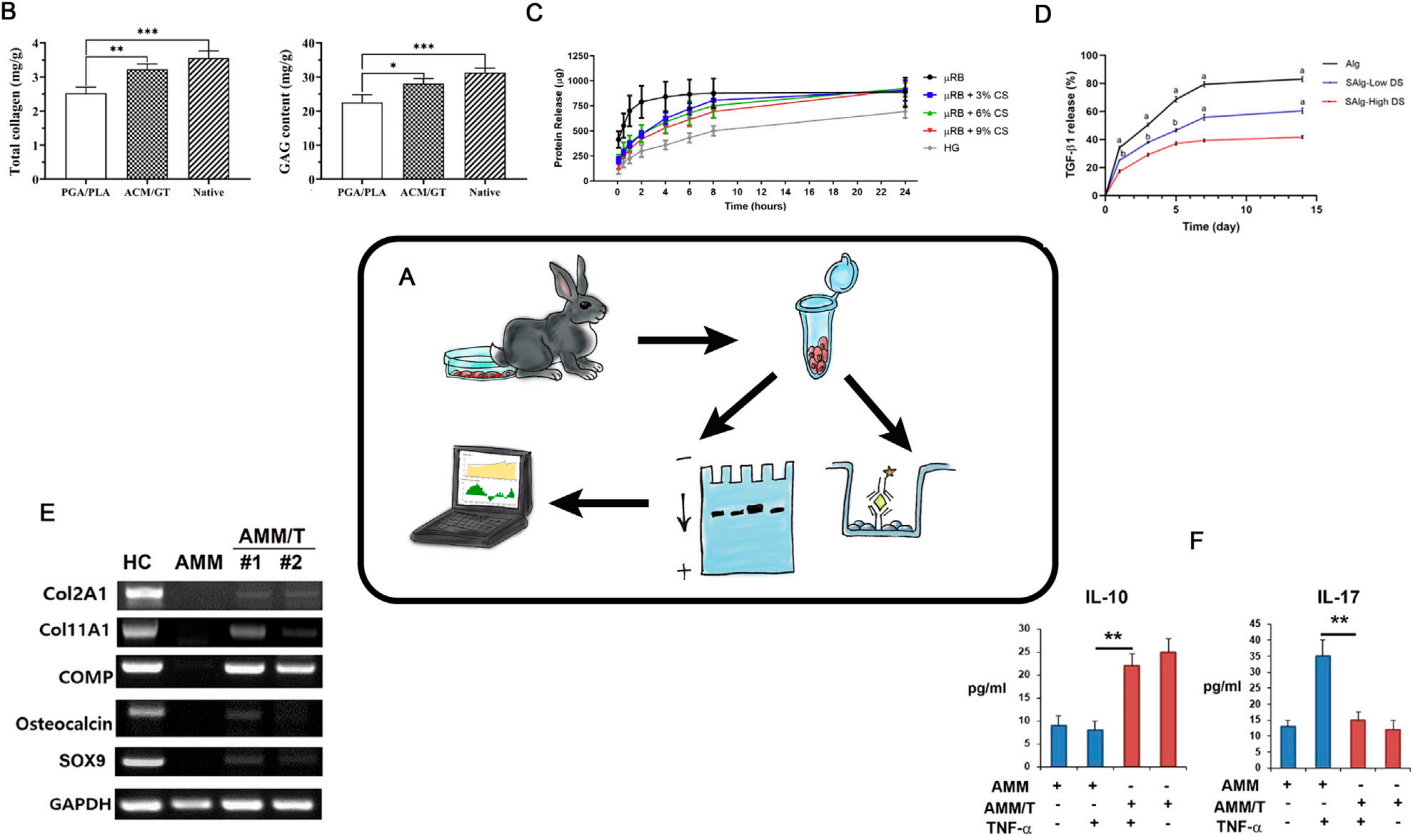
Protein Analysis in TEHC **(A)** – Schematic representation of the protein analysis in hyaline cartilage tissue engineering. **(B)**– Total collagen, GAG content. Taken from the article (Jia et al., 2021). **(C)**– Protein elution profile of BSA from all scaffolds over 24 h (n = 5/group). Taken from the article (Rogan et al., 2020). **(D)**– Percent cumulative transforming growth factor- β1 (TGF-β1) release from hydrogels over 14. Taken from the article (Payam et al., 2021). **(E)**– Western blot. Protein synthesis level. Taken from the article (Chae et al., 2021). **(F)**- ELISA results showed that coculture with AMM/T resulted in significantly higher IL-10 and lower IL-17A levels in cell culture supernatant compared with AMMs. Taken from the article (Chae et al., 2021).

The primary functional role in HC is performed by ECM proteins. Assessing the protein composition of the regenerate or developed CECs is a key analytical task for evaluating the efficacy of tissue engineering of hyaline cartilage. Methods for studying protein composition can be divided into semi-quantitative (“presence or absence” of specific proteins) and quantitative (determining the amount of protein per mass or volume unit) (Figure 5A).

Currently, polyacrylamide gels (PAGEs) are widely used to separate protein (Burnette, 1981). After the protein separation, staining and semi-quantitative assessment can be performed with immunoblotting (i.e., Western blotting) (Alwine et al., 1977; Hawkes et al., 1982) (Figure 5E). The determination of the protein here relies on the specific interaction between antigen and antibody. Currently, commercially available kits allow for the semi-quantitative assessment of specific protein release even at the *in vitro* stage (Figures 5B–D,F). Depending on the modifications and the use of different cell-engineering constructs, the content of specific ECM proteins can be evaluated both *in vitro* and *in vivo* with precision down to nanograms.

In HC defects repairing studies, the selection and evaluation of cell-engineering construct parameters are impossible without the analysis of the protein composition. Researchers use semi-quantitative methods as the PAGE and immunoblotting, but more commonly (and preferably), highly accurate quantitative methods based on ELISA (Enzyme-linked immunosorbent assay) should be applied. ELISA can precisely determinate the absolute ECM protein amounts in the given CECs, providing critical insights into the effectiveness of the entire methodology at both intermediate and final stages of research. For tissue engineering of hyaline cartilage, it is the optimal protein composition that is important for the formation of a regenerate resistant to mechanical stress. In studies where cell culture modification is used, it is a prerequisite to confirm this by analysing the changes in protein composition. These analyses are simple, economically accessible methods for the evaluation of the quantitative protein composition in both *in vitro* and *in vivo* experiments.

## Biosensors

A new and dynamically developing area is the use of biosensors for the determination of protein composition. To date, existing methods for detection of specific proteins are mainly based on ELISA assay. However, ELISA assays have the following disadvantages, such as lack of accuracy in detecting small amounts of proteins, false positive results, and significant analysis duration. Such methods cannot be used for early diagnosis of diseases and/or for detection of small amounts of proteins. A biosensor is a device that provides an electrical pulse reading from a test preparation depending on the concentration of a particular protein. For the current period biosensors for detection and sensing are fundamentally divided into are electrochemical, optical, Quartz crystal microbalance (QCM), molecular and wearable biosensors (Wang et al., 2010). The advantages of such biosensors are as follows: ease of use, accuracy of measurements, possibility of mass production, low cost (when they are put into mass production). This new direction is dynamically developing; however, it has not yet received mass use due to the complexity of development and design. An example of such a biosensor for the detection of an early marker of osteoarthritis was given in a clinical article, where a measurement accuracy of 0.2 * 10^−18^ per ml of solution was reported (Lv et al., 2024), (Ahn et al., 2011). For some diseases associated with OA where additional detection accuracy is required (such as juvenile idiopathic arthritis, for example), such biosensors may be the only solution (Rodovalho et al., 2018). Thus, these findings are being actively applied already in clinical practice and allow for precision assessment of parameters such as protein composition, etc. We could not find works that combine classical tissue engineering experiments on animals and the use of biosensors; however, this is apparently a matter for the near future.

## Cell viability analysis

**FIGURE 6:**
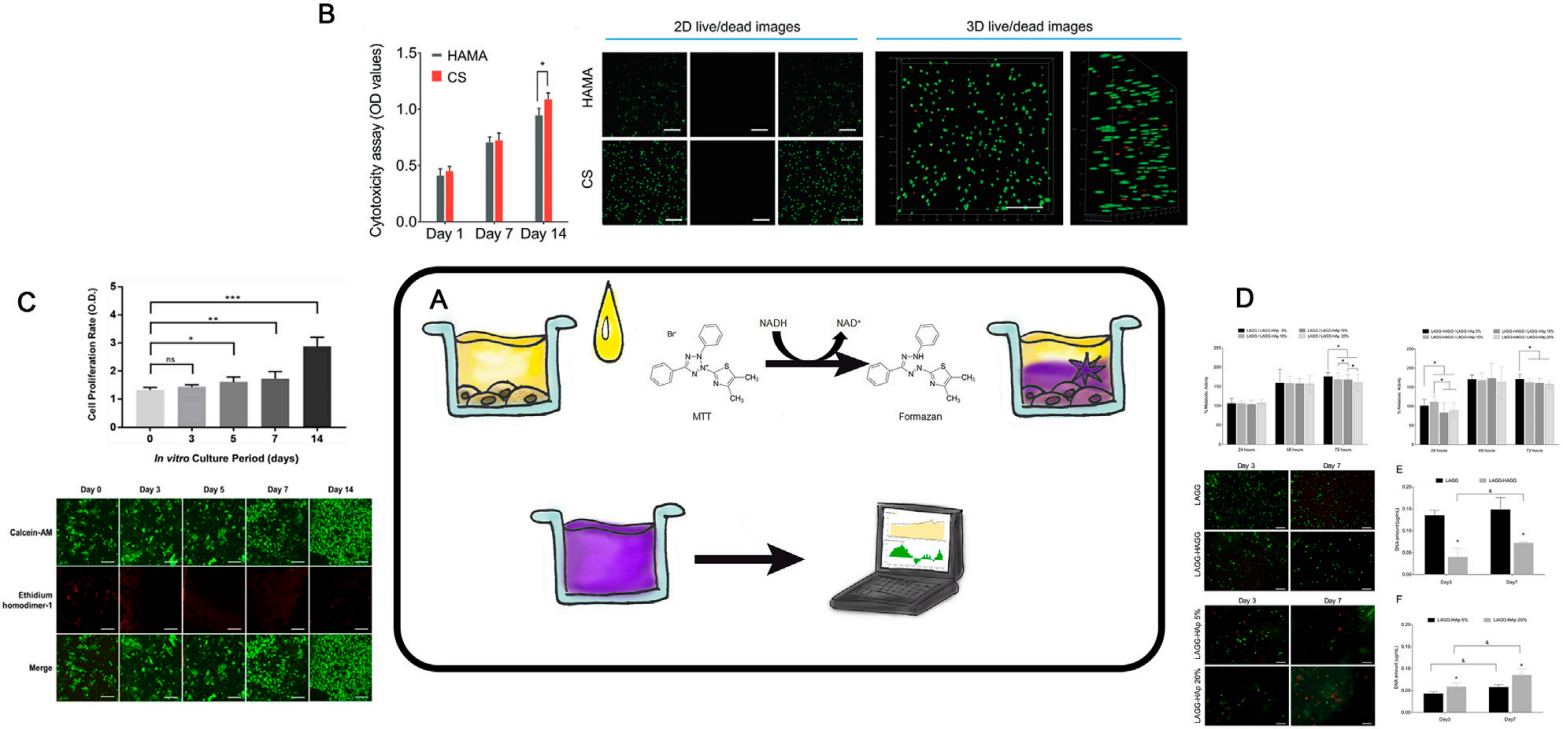
Cell Viability Analysis **(A)**– Schematic representation of the MTT assay procedure. **(B)**– CCK8 assay of hBMSCs encapsulated in the indicated hydrogels and 2D live/dead staining images of hBMSCs after encapsulation in the HAMA and CS hydrogels for 7 days. Taken from the article (Liu et al., 2020). **(C)**– In vitro Cell proliferation and viability assay for human chondrocyte in Silk-GMA hydrogel. CCK 8 assay for cell proliferation rate increasedaccording to culture period, gradually. Data are shown as the mean ± SD (*p < 0.05, **p < 0.005 and ***p > 0.0005, respectively). Confocal microscopic images for Live & Dead assay with Calcein-AM (live cells, green fluorescence) and ethidium homodimer-1 (dead cells, red fluorescence) staining showed that human chondrocytes were proliferated well in 30% of Silk-GMA hydrogel up to 2 weeks cultivation (Scale bar = 500 μm). Taken from the article (Hong et al., 2020). **(D)**- In-vitro screening of cytotoxicity of BHC. Metabolic activity of L929 exposed to LAGG/LAGG-HAp and LAGG-HAGG/LAGG-HAp extracts respectively, for a period of 72 h. (*) Indicates a significant difference between groups for the same time point (p < 0.05). Live/dead staining by Calcein AM/PI, of chondrocytes within LAGG and LAGG-HAGG and osteoblasts within LAGG-HAp 5% and 20%. OC-derived cells were cultured for 7 days. Scale bar represents 200 m. Proliferation of the embedded chondrocytes and osteoblasts within respective formulations up to 7 days. (*) Indicates a significant difference between groups for the same time point and (&) indicates a significant difference between time points for the same formulation (p < 0.05). Taken from the article (Pereira et al., 2018).

An essential stage in tissue engineering of hyaline cartilage involves a development of cell-engineering construct composed of a cell culture and a biodegradable scaffold. However, achieving a proliferating cell culture on the surface or within the scaffold remains a challenging technological task. Therefore, evaluating the viability of cells cultured in 3D conditions is a critical and necessary intermediate step in modern tissue engineering of hyaline cartilage (Figure 6A).

The simplest and the most accessible method to recognize the potential cytotoxic effects of a scaffold on the living cells involves an analysis of exudates after the scaffold co-incubation. During the incubation, scaffold components may leach into the media, negatively affecting cell viability (Lee et al., 2020a). The most commonly, the MTT assay is used. It measures the ability of NADPH- dependent cellular oxidoreductases to reduce the tetrazolium dye 3-(4,5-dimethylthiazol-2-yl)-2,5-diphenyl-tetrazolium bromide into insoluble formazan (Figure 6B) with can later be detected spectrophotometrically. For example, Gögele evaluated the potential cytotoxicity of glass-containing polylactide-glycolide copolymer scaffolds using the MTT assay (Gögele et al., 2022). The most accurate method is to analyze the viability of cells directly in contact with the scaffold, often using again the modified MTT assay. Haghighi (Paniz and Shamloo, 2021) used it to evaluate the viability of chondrocytes cultured in silk fibroin-based scaffolds. This method has limitations, such as the potential sorption of formazan by the scaffold, which may decrease the optical density of the analyzed solution.

The MTT assay also enables comparative analysis of how many viable cell within the scaffold (Vinod et al., 2019). Cell viability in the gels was determined via gel staining, which is only possible with optically transparent gels. An alternative to the MTT assay is the MTS assay (Payam et al., 2021), which works on a similar principle. Sun et al. assessed chondrocytes in scaffolds based on methacrylated polyethylene glycol using MTS, analyzing optical density at 492 nm after a 4-h incubation (Sun et al., 2015).

Calcein staining is another accurate and specific method to determine the cell viability for both cultured on the scaffold surface and within it (limited to optically transparent gels) (Liu et al., 2020; Vinod et al., 2019) (Figures 6C,D). This reagent can penetrate the cell membrane of living cells, where intracellular esterases cleave its acetoxymethyl group, causing calcein to fluoresce in the green spectrum. Simultaneously, propidium iodide can be added to identify dead cells. For instance, using fluorescence microscopy, Gögele and authors assessed the viability of chondrocytes on the surface of a polylactide-glycolide copolymer-based scaffold after calcein and propidium iodide staining (Gögele et al., 2022). The rate of cell viability, expressed as the ratio of alive cells to the total number of cells (both live and dead), was analyzed in Acar’s work with polymer (Karabıyık Acar et al., 2021), as well as in a series of studies involving gels (Hong et al., 2020; Pereira et al., 2018; Oyadomari et al., 2021). The transparency of chitosan- and hyaluronic acid-based gels allows for evaluating of chondrocytes not only on the scaffold surface but also throughout the entire gel depth. Sun and colleagues in their study assessed cell viability using calcein staining (Sun et al., 2015). Another solution suitable for FC and microscopy is the commercial ViaQuant™ Far-Red Dead Cell Staining Kit, designed to distinguish live and dead cells. This kit relies on a reaction of a fluorescent dye with cellular amines and emits light in far-red range, being applicable for *in vivo* studies (He et al., 2021). For the effective implantation of cell-engineering constructs and regeneration of the modelled defect, it is important to achieve minimal cytotoxicity of the scaffold for the cell culture used and to use methods to assess cell viability at the *in vitro* stage. This type of method is a simple, cost-effective way to obtain numerical data on cytotoxicity and cell viability in combination with the scaffold. Currently, evaluating the precise viability of cell cultures within opaque 3D objects remains a complex and unresolved challenge. Traditional methods are not fully reliable in such cases and no universal method has been developed for this purpose.

## Strength studies

**FIGURE 7:**
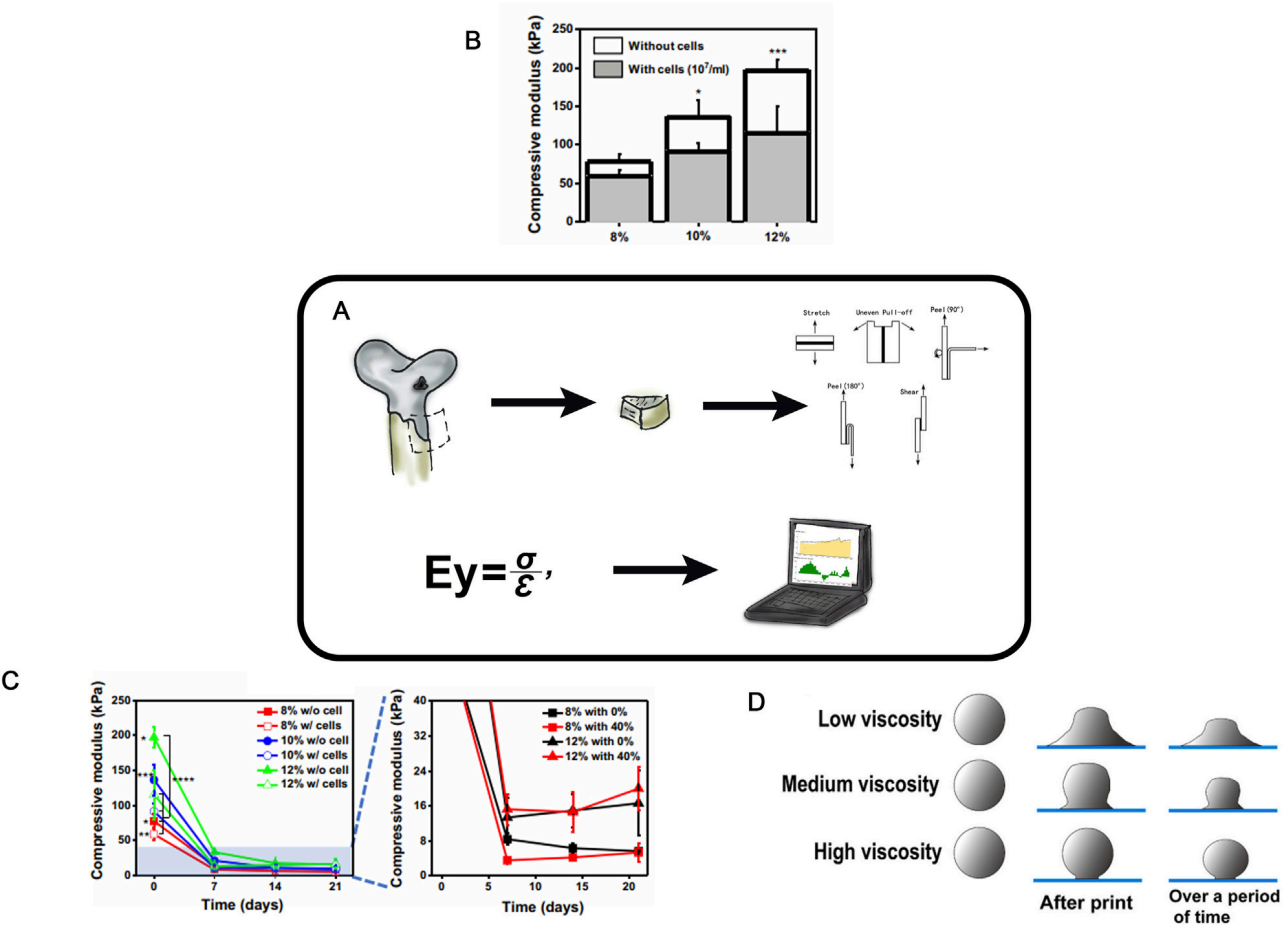
Strength Studies in TEHC **(A)**– Schematic of strength testing of cellular-engineered constructs **(B)**- Compressive modulus of PEG/OMA hydrogels (8, 10, and 12% PEG/OMA) with or without cells on day 0 (n = 5) (One-way ANOVA with Tukey’s significant difference post hoc test; *P < 0.05 and ***P < 0.005 compared with 8% without cells). Taken from the article (Lee et al., 2020b). **(C)**- Time profile of hydrogel degradation without compression for 21 days (n = 5). (One-way ANOVA with Tukey’s significant difference post hoc test; *P < 0.05 compared with 10% without cell group, **P < 0.05 compared with 12% with cell group, ***P < 0.005 compared with 8% with cells, and ****P < 0.005 compared with 12% without cell group at day 0.) Taken from the article (Lee et al., 2020b). **(D)**- Schematic diagram of MEW electrospinning with different viscosities. Taken from the article (Han et al., 2021a).

Due to its high content of proteoglycans and collagen fibrils, hyaline cartilage is characterized by high strength, resilience, elasticity, and density. Thus, it is enabling to withstand significant loads during the body’s physiological activity (Liu and Karan, 2021). A key task for the full recovery is to create an implant with physical and mechanical properties similar to those of hyaline cartilage. Therefore, during the development of cell-engineering constructs, it is critically important to evaluate their mechanical properties, specifically the ability of the experimental construct to endure mechanical loads (Galarraga et al., 2021) both *in vitro* and *in vivo* (Figure 7A).

There are certain examples of dynamic changes in the physical properties of cell-engineering constructs depending on their composition (cellular, gel-based, etc.) (Figures 7B,C). The creation of mechanically resilient cell-engineering constructs, for instance, from gels, can be achieved by increasing the polymer concentration in the scaffold or enhancing crosslinking via higher levels of a crosslinking agent (Loebel et al., 2020; Stephanie and Anseth, 2002), as well as by the formation of composite hydrogels with polymers of diverse chemical structures and mechanical properties (Hashemibeni et al., 2020; Ciardulli et al., 2020; Ali et al., 2020). These solutions increase the mechanical stability of the scaffold by enhancing its density and, typically, reducing the pore size. However, such dense constructs can impair cell migration into the scaffold, thereby reducing cell viability (Liu et al., 2022b; Han et al., 2021b).

Hoenig et al. assessed the effect of subchondral bone permeability on the properties of CEC (Hoenig et al., 2013). Native cartilage-bone cylinders retrieved from pigs were cultured for 2 weeks in a bioreactor under a mechanical load, with and without restricted bone permeability. The Young’s modulus and stiffness of each cartilage sample were determined using an unconfined compression test consisting of five sequential deformation loads and six loading cycles.

Middendorf investigated the complex mechanical behavior, function, and temporal changes in cultured *in vitro* tissue engineering of hyaline cartilage via compression, friction, and shear tests (Middendorf et al., 2017). The compression and friction tests revealed improved properties of the construct with prolonged culture time. The elasticity correlated with glycosaminoglycan (GAG) content, while the improved friction coefficients were associated with increased lubrication of the construct surface (Gregorio et al., 2019). The elasticity or compressive modulus of HC scaffolds were evaluated via uniaxial compression tests (Lee et al., 2020b). Typically, cylindrical samples are prepared under physiological conditions in a swollen state to mimic *in vivo* situation. The compressive modulus is calculated as the slope of the stress-strain curve during deformation, and can be improved by modifying the cell-engineering construct structure or components (Figure 7B). A decrease in scaffold stiffness—and thus a reduction in the compressive modulus—can be caused by intra-scaffold cell culturing (Figure 7C).

A precise control of polymer viscosity, elasticity, and phase transitions is particularly important during the scaffold formation (Gregorio et al., 2019). Phase transitions can be evaluated by the rheological properties with a rheometer (Figure 7D) (Han et al., 2021a). Polymer rheology can indicate mechanical resilience as well as confirms an increased crosslinking in hydrogels.

By using different scaffolds in combination with different cell cultures (possibly pre-modified), researchers obtain different physical and mechanical properties of cell-engineering construct. In order to analyze the influence of each of these factors on the final parameters of the resulting objects, it is necessary to apply just this set of techniques. 

The primary goal is the quantitative analysis of the mechanical parameters of developed cell-engineering construct to create optimized constructs for HC repair and to study the effects of various cell-engineering construct parameters (or cell modifications) on these properties. Specialized equipment is required for the mechanical tests, but the experiments themselves are simple and economically accessible.

## Micro-computed tomography (MCT)

**FIGURE 8:**
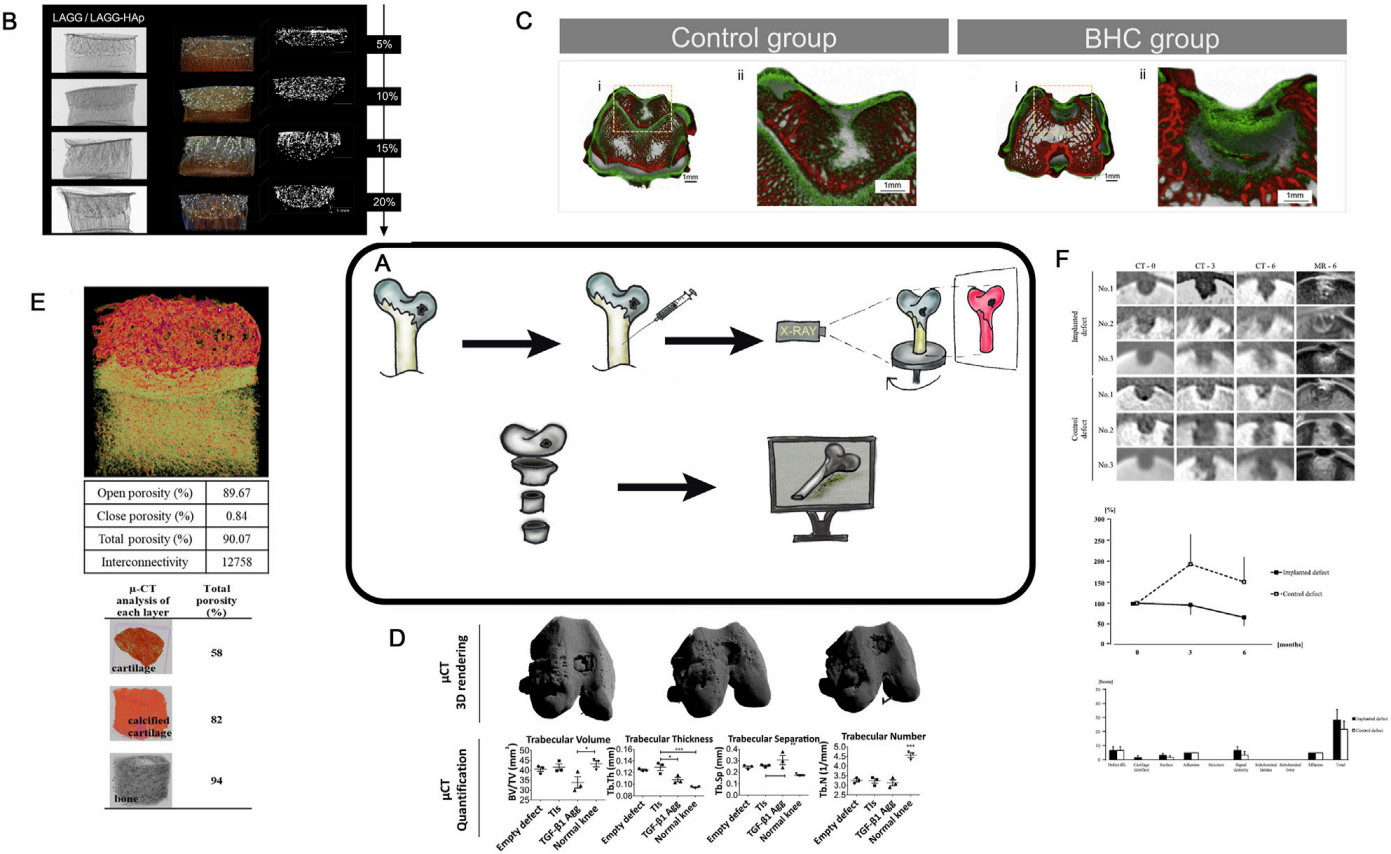
**(A)**– Schematic diagram of MCT approach. **(B)**- MCT analysis of different experimental CEC. Scale bar represents 1 mm. Taken from the article [71=60]. **(C)**- MCT micrographs of the explanted rabbit’s knees at 4 weeks after surgery for Control group and Experimental group. 3D explant images showing hard tissue (bone-like tissue , red colour) and soft tissue (cartilagelike tissue & hydrogel, green colour). Taken from the article (Pereira et al., 2018). **(D)**- MCT 3D rendering showed subchondral bone regeneration at the osteochondral defect site. Quantification and characterization of new subchondral bone formation at the defect site was performed by analyzing a region of interest 1.5 mm diameter × 1 mm depth. n = 3 animals per condition. One-way ANOVA followed by Tukey’s multiple comparison test was used to analyze the results. *P < 0.05, **P < 0.01, ***P < 0.001. Taken from the article (Mendes et al., 2018). **(E)**- MCT representation and porosity evaluation of multi-layered scaffold and each distinct layer. The red dots in the multi-layered view represent the nHA particles. Taken from the article (Korpayev et al., 2020). **(F)**- MCT assessment of osteochondral defects. MCT images show one cross-section of the multi-planar reconstruction images at one (CT-0), three (CT-3), and six (CT-6) months after the surgery in No. 1, No. 2, and No. 3, and MR images (MR-6) show the images corresponding to the MCT images at 6 months after surgery. Line graph shows the averages of RV (radiolucent volume) percentages at the third and sixth months against those at month zero in both defects. Bar graph shows the averages of the items in the Modified 2D-MOCART scores based on the images of MR-6 Taken from the article (Murata et al., 2022).

MCT, first developed in the early 1980s (Flannery et al., 1987; Feldkamp et al., 1989) is an X-ray imaging method that enables the acquisition of 3D images of objects (Figure 8A). It is now actively used in tissue engineering of hyaline cartilage research, both in experimental *in vivo* research (Ki et al., 2018; Batiste et al., 2004), and in assessment of scaffold and construct characteristics (Tsai et al., 2015; Bertoldi et al., 2011; Liao et al., 2015; Swieszkowski et al., 2007) and morphological changes in articular cartilage under physical stress (Rapagna et al., 2022) and during aging (Moncayo-Donoso et al., 2019). MCT provides a detailed imaging of tissue engineering of hyaline cartilage (Figure 8B) in animal models (Figure 8C) with high resolution, making it particularly suitable for the small size experimental animals. Another important feature of the technique is an opportunity to create 3D models of the study area (Figure 8E).

MCT enables both the visualization and quantitative assessment of bone and cartilage 3D tissue formation during the implantation of tissue engineering of hyaline cartilage in animal models (Murata et al., 2022; Duke et al., 2009; Jaecques et al., 2004; Hutmacher, 2005). This technique helps to examine samples *in vitro, in vivo*, and *ex vivo* (Figures 8B–E). Saey Tuan Ho et al. compared MCT with other methods for characterizing scaffolds in TE and highlighted several advantages of the technique (Ho and Hutmacher, 2006). These include the ability to assess scaffold porosity, its interconnections, surface, and permeability (Figures 8D–F). MCT was used to evaluate and visualize different regions of a chitosan- and collagen-based construct after implantation (Figure 8E) (Korpayev et al., 2020). In another study (Swieszkowski et al., 2007), MCT was employed to evaluate similar parameters for biphasic scaffolds composed of polycaprolactone and fibrin, as well as polycaprolactone and tricalcium phosphate. The authors seeded cells into these constructs, created defects, implanted the constructs and evaluated the recovery with MCT. Mendes et al. used MCT to compare various constructs, enabling both visual and quantitative assessment of the cell density in regenerative regions (Figure 8E) (Mendes et al., 2018).

For a more detailed visualization of soft tissues such as cartilage, MCT can be combined with Equilibrium Partitioning of Ionic Contrast agents (EPIC- MCT) (Frank et al., 2005). Recently, contrast agents such as iothalamate (Cysto-Conray^®^ II) (Entezari et al., 2014) and ioxaglate (Hexabrix^®^) (Kerckhofs et al., 2014) have been developed, enabling MCT imaging of unmineralized cartilage due to the charged nature of the cartilage ECM. Xiao-Fei Li used EPIC-MCT to confirm age-related changes in sulfated glycosaminoglycan (sGAG) to describe cartilage degeneration (Li et al., 2015). Palmer et al. demonstrated that EPIC- MCT is a quantitative, non-biased, noninvasive, and highly accurate method to assess cartilage composition and 3D morphology in cartilage degeneration studies (Palmer et al., 2006).

MCT is a high-precision, noninvasive approach for quantitative evaluation of regenerative changes and *in vivo* morphology in studies of cartilage degeneration and repair after experimental interventions (both *in vivo* and *in vitro*). This method is simple and cost-effective, but requires specialized equipment, especially for the cell-engineering construct analysis. MCT helps to quantify the defect progression and replacement in HC of animal models with a high resolution (up to 6 µm). The simultaneous visualization of soft tissues can be achieved via contrast enhancement. MCT holds a great potential for further application, as it enables noninvasive computation of numerous numerical parameters related to the internal structure of constructs. By date, there are certain steps in development of automated multiparametric analysis protocols for constructs using MCT (Mendes et al., 2018), including the use of AI to analyze such regenerative changes has become widespread.

## Scanning electron microscopy (SEM)

**FIGURE 9:**
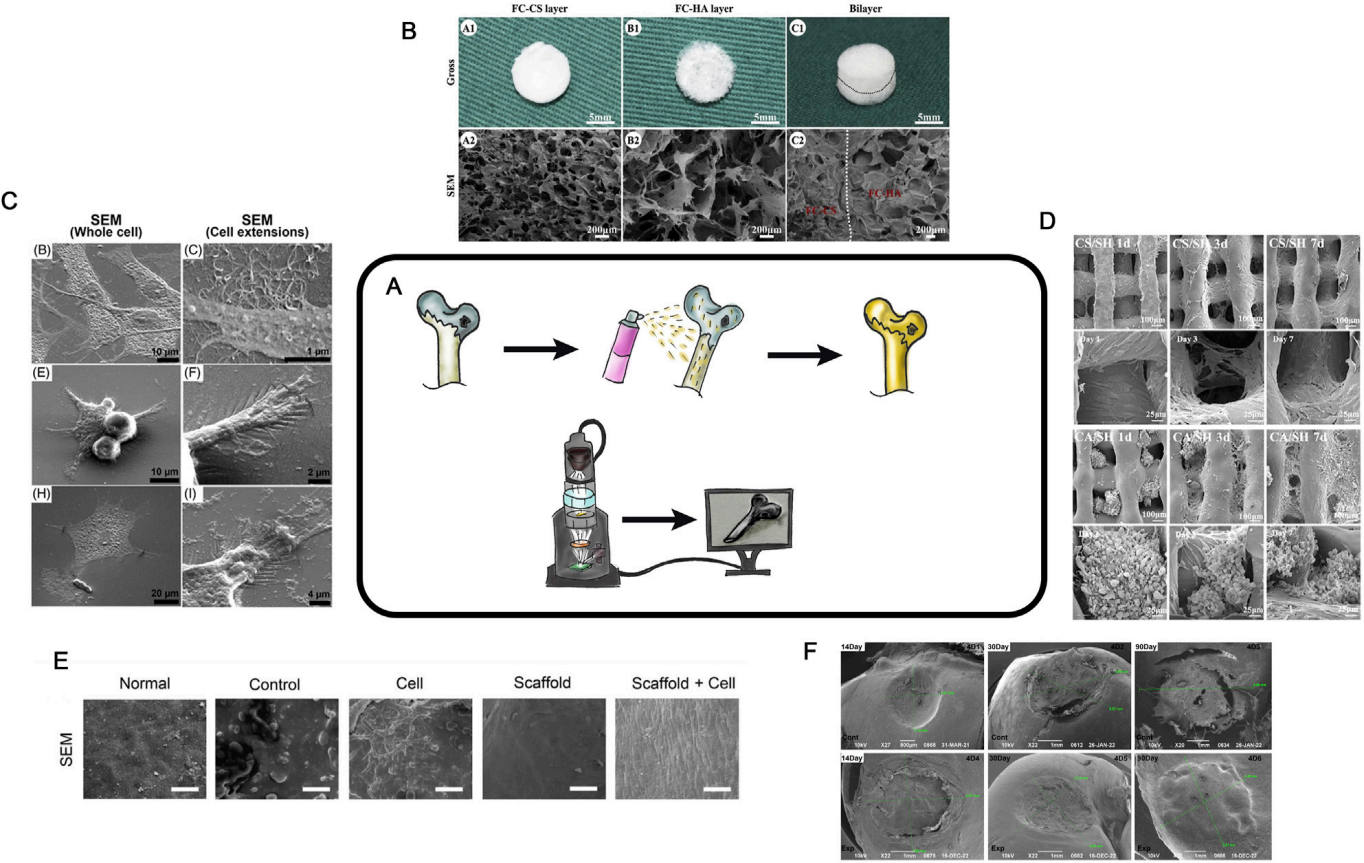
SEM in TEHC **(A)**– Schematic diagram of the SEM method. **(B)**– Macroscopic and microscopic views of fish collagen chondroitin sulfate and fish collagen hydroxyapatite scaffolds. The gross (A1-C1), SEM (A2-C2). Taken from the article Zhou et al. (2020). **(C)**– SEM micrographs taken after 3 days of culture (middle and right) of chondrocytes grown in different groups. (Katrín et al., 2022) **(D)**– SEM of different groups at 1, 3 and 7 days. Taken from the article Li et al. (2021) **(E)**– SEM images of Surface area, normal group is smooth, MF and cell group was smoother than those of the other three groups, and in scaffold cell groups the cells were attached to scaffold in cartilage tissue is visualized (scale bar = 30 μm). Taken from the article Dadgar et al. (2021) **(F)**- SEM images of Surface area, measurement of resulting hyaline cartilage defects. (authors unpublished data).

SEM visualizes of the surface of a sample up to 10 nm range (Figure 9A). The potential for applying SEM to HC research was first reported in 1971 (Clarke, 1971). The sample surface is coated with a conductive layer and then placed in an electromagnetic field. By analyzing the deflection of the electron beam generated by an electron gun, it becomes possible to visualize the surface.

SEM is now widely used for examining scaffold porosity (Figure 9B) (Zhou et al., 2020). It can also be employed to analyze individual cells on the surface (Figure 9C) (Katrín et al., 2022). After cell seeding and the formation of CEC, SEM enables the analysis of cell proliferation, migration, and distribution both on the surface and within the scaffold over various cultivation periods (Figures 9D,E). During *in vivo* studies, SEM can be used to evaluate the regeneration area after CEC implantation. It allows assessing of the surface structure, the defected or regenerated area, the contact zone of the scaffold and surrounding tissues at the defect margin, and provides data for scoring systems (e.g., International Cartilage Repair Society - ICRS) (Figure 9F) (Zheng et al., 2019). This technique enables a detailed visualization of the structures. The advantages of SEM include the simplicity of sample preparation (typically limited to drying and dehydration), the ability to obtain numerical data (e.g., pore sizes, sample or defect dimensions, cell distribution on the surface, and surface layer structure), and its high-resolution imaging. This technique is cost-effective, but requires a direct scanning electron microscope. SEM is not strictly necessary, but allows good visualization of the area of interest at all stages.

## FDA-approved methods

The significant progress made in experimental tissue engineering of hyaline cartilage implies the introduction of similar techniques, with some time lag, into clinical practice. The set of techniques for analyzing clinical efficacy differs somewhat between experimental and clinical practice. For logical and understandable reasons, non-invasive or minimally invasive techniques take precedence in clinical practice. In clinical practice mainly used are: MRI (CT) diagnosis, X-rays, questionnaires and arthroscopy in rare cases. Protein assay or RT-PCR methods are sometimes used and biosensors have begun to be introduced to provide a wide range of data. The authors are aware of only a few studies involving the use of histological (invasive) methods of analysis. SEM and histology are not used at all. Flow cytometry is also not investigated directly from the area of interest (at the site of cell-engineering construct transplantation). We believe that invasive methods of analysis should ideally be used at the stage of cell-engineering construct preparation to confirm its safety and efficacy, whereas after implantation only minimally invasive methods should be used to minimize additional trauma to the patient.

## Conclusion

**FIGURE 10:**
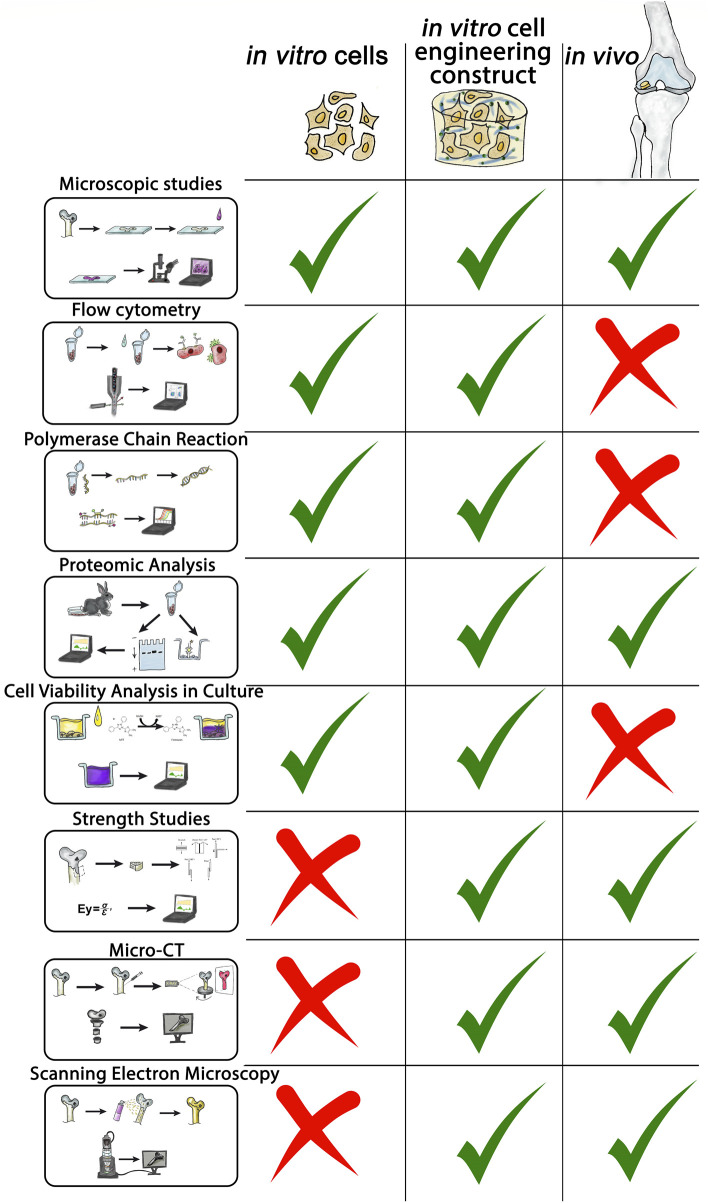
Methods in TEHC analysis.

Experimental tissue engineering of hyaline cartilage is a technologically complex field that requires researchers to apply a wide range of methods, both during the experimental phase and in the evaluation of results. In this work, we highlighted the main methods used to evaluate the experimental effectiveness of tissue engineering of hyaline cartilage (Figure 10).

The listed and analyzed methodologies allow creating of a summary table outlining their applications, advantages, and limitations (Table 1). The relationship between the various methods is shown additionally in the attached filе (Supplementary Figure S1).

**TABLE 1:** Comparative characteristics of methods for analyzing experimental TEHC results.

Method	Applicability	Invasiveness	Difficulty of use	Economic accessibility	Clinical application	Data type qualitative(Q), semi-quantitative (SQ)	What it evaluates
Microscopy	*in vitro\* *in vivo*	Yes	***	**	No	Q/SQ	Cartilage regeneration, structure of tissue
SEM	*in vitro\* *in vivo*	Yes	**	**	No	Q/SQ	3D structure
Micro-CT	*in vitro\* *in vivo*	No	**	***	Yes	Q/SQ	3D structure
PCR	*in vitro\* *in vivo*	Yes	*	*	Yes	Q	Gene expression
Protein analysis	*in vitro\* *in vivo*	Yes	*	**	Yes	Q/SQ	Protein synthesis
MTT	*in vitro*	Yes	**	*	No	Q	Cytotoxicity, effect on cell metabolism
Mechanical testing	*in vitro\in vivo*	Yes	*	***	No	Q	Physical properties of samples
Flow cytometry	*in vitro*	Yes	*	**	No	Q	Surface cell markers

All the employed methods have become quantitative, suitable for comparative analysis across studies. Among these methods, histological analysis remains particularly important. In our opinion, histology is an essential reference method to be compared to all other techniques. Modern tissue engineering of hyaline cartilage studies employ a wide variety of techniques and require access to advanced equipment or close collaboration of multiple laboratories to work in one direction. Thus, a defining feature of contemporary experimental tissue engineering of hyaline cartilage studies is the comprehensive application of all (or nearly all) of the aforementioned methods for effective analysis.

## Abbreviations

HC, Hyaline cartilage; TEHC, Tissue engineering of hyaline cartilage; CEC, Cell-engineered construct; ECM, Extracellular matrix; ICRS, International Cartilage Repair Society grading system; FC, Flow cytometry; ASC, adipose stem cell; FACS, fluorescence-activated cell sorting; RT-PCR, Reverse transcription polymerase chain reaction; PCR, Polymerase chain reaction; BMSCs, Bone marrow-derived stem cells; GDF5, Growth differentiation factor-5; hMSCs, Human mesenchymal multipotent stromal cells; IVFC, *In vivo* flow cytometry; ELISA, Enzyme-linked immunosorbent assay; GAG, Glycosaminoglycans; MCT, Micro-computed tomography; EPIC, equilibrium partitioning of ionic contrast agents; SEM, Scanning electron microscopy; ICRS, International Cartilage Repair Society.
