# Supplementary figures and images for: Modern experimental methods for assessing the effectiveness of tissue-engineered products for hyaline cartilage regeneration
Source: Front Bioeng Biotechnol. 2025 Jul 21;13:1595116. doi: 10.3389/fbioe.2025.1595116 (PMC12318997; doi:10.3389/fbioe.2025.1595116)

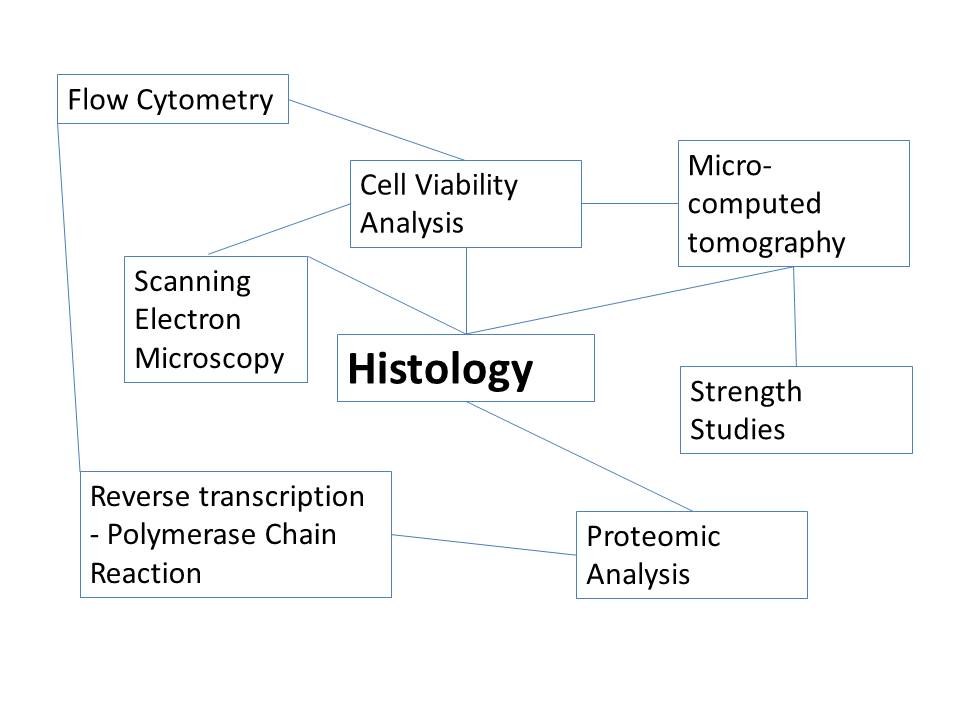

Supplement: Supplementary file 1 [file Image1.jpeg]
